# Microarray analysis distinguishes differential gene expression patterns from large and small colony *Thymidine kinase *mutants of L5178Y mouse lymphoma cells

**DOI:** 10.1186/1471-2105-7-S2-S9

**Published:** 2006-09-26

**Authors:** Tao Han, Jianyong Wang, Weida Tong, Martha M Moore, James C Fuscoe, Tao Chen

**Affiliations:** 1 Division of Systems Toxicology, National Center for Toxicological Research, U.S. FDA, Jefferson, AR, USA; 2 Division of Genetic and Reproductive Toxicology, National Center for Toxicological Research, U.S. FDA, Jefferson, AR, USA

## Abstract

**Background:**

The *Thymidine kinase *(*Tk*) mutants generated from the widely used L5178Y mouse lymphoma assay fall into two categories, small colony and large colony. Cells from the large colonies grow at a normal rate while cells from the small colonies grow slower than normal. The relative proportion of large and small colonies after mutagen treatment is associated with a mutagen's ability to induce point mutations and/or chromosomal mutations. The molecular distinction between large and small colony mutants, however, is not clear.

**Results:**

To gain insights into the underlying mechanisms responsible for the mutant colony phenotype, microarray gene expression analysis was carried out on 4 small and 4 large colony *Tk *mutant samples. NCTR-fabricated long-oligonucleotide microarrays of 20,000 mouse genes were used in a two-color reference design experiment. The data were analyzed within ArrayTrack software that was developed at the NCTR. Principal component analysis and hierarchical clustering of the gene expression profiles showed that the samples were clearly separated into two groups based on their colony size phenotypes. The Welch T-test was used for determining significant changes in gene expression between the large and small colony groups and 90 genes whose expression was significantly altered were identified (*p *< 0.01; fold change > 1.5). Using Ingenuity Pathways Analysis (IPA), 50 out of the 90 significant genes were found in the IPA database and mapped to four networks associated with cell growth. Eleven percent of the 90 significant genes were located on chromosome 11 where the *Tk *gene resides while only 5.6% of the genes on the microarrays mapped to chromosome 11. All of the chromosome 11 significant genes were expressed at a higher level in the small colony mutants compared to the large colony mutants. Also, most of the significant genes located on chromosome 11 were disproportionally concentrated on the distal end of chromosome 11 where the *Tk *mutations occurred.

**Conclusion:**

The results indicate that microarray analysis can define cellular phenotypes and identify genes that are related to the colony size phenotypes. The findings suggest that genes in the DNA segment altered by the *Tk *mutations were significantly up-regulated in the small colony mutants, but not in the large colony mutants, leading to differential expression of a set of growth regulation genes that are related to cell apoptosis and other cellular functions related to the restriction of cell growth.

## Background

The mouse lymphoma assay (MLA) is used internationally for regulatory decision-making and it is the mammalian *in vitro *gene mutation assay preferred by the U.S. FDA, the U.S. EPA, and the International Committee on Harmonization (including the European, Japanese and U.S. pharmaceutical companies and regulatory agencies) [1-3]. The MLA is conducted using an L5178Y cell line that is heterozygous for the *Tk *gene. The assay detects forward mutation of the wild-type *Tk *allele (*Tk*1b) located on mouse chromosome 11 [4]. In this assay, *Tk*-deficient (*Tk*^-/- ^or *Tk*^0/-^) mutants of the L5178Y/*Tk*^+/- ^mouse lymphoma cells are selected by the pyrimidine analog trifluorothymidine (TFT) because TFT inhibits division of the *Tk *competent (*Tk*^+/-^) cells that are capable of incorporating TFT into the DNA. The mutant cells cannot incorporate TFT into their DNA because of the *Tk *gene deficiency. Therefore, the mutants can grow and develop into colonies in the selective growth medium while the *Tk*-competent cells are growth arrested and do not divide.

A striking feature of the *Tk *mutant colonies recovered in the MLA is the presence of two size classes of mutants. Immediately following their isolation, the cells in the large colonies grow at a normal rate, while cells in the small colonies grow slowly. The relative frequency of the two colony classes is mutagen dependent. Generally, clastogens induce more small colony mutants while point mutagens induce more large colony mutants [5-8]. It should be noted that many chemicals induce both small and large colony mutants.

It is important to obtain definitive information concerning the underlying molecular basis for the small and large colony mutant phenotype. This can be particularly important in a regulatory context. There is increasing interest in distinguishing between chemicals that cause point mutations and those that cause chromosomal mutants. The small and large colony *Tk *mutant phenotype was identified more than 30 years ago and there have been several hypotheses proposed to explain the difference between these two mutant types. The first one suggested that the small-colony mutants result from large scale damage to the chromosome 11b on which the *Tk*^+ ^allele resides while large-colony mutants result from mutational events affecting the expression of only the *Tk *gene [9-11]. This hypothesis was expanded to state that small colony mutants are the consequence of intergenic lesions affecting the *Tk *gene and other putative growth control gene(s) that may or may not be on chromosome 11. Large colony mutants are the consequence of either intragenic lesions limited to the *Tk *gene or intergenic lesions that do not affect the growth control gene [12,13]. So far, the growth control gene or genes have not been identified. Further analysis using microsatellite markers on chromosome 11 demonstrated that both large and small colony mutants can apparently have relatively large alterations of chromosome 11b [14,15]. Also, it is clear that the entire chromosome 11b is lost in some large colony mutants [15]. Another possible hypothesis, demonstrated in other cell types, invokes a process of chromosome damage and repair. In this model, a cell with chromosome damage would suffer arrested growth until the cell repairs the damage [16,17]. None of these hypotheses, however, can fully explain the difference between the small and large colony phenotypes because the molecular analysis of mutants (primarily microsatellite analysis) does not reveal a clear cut distinction in the degree of "damage" between small and large colony mutants. That is, the fundamental mechanistic difference(s) between the small and large colony mutant phenotypes has not been elucidated using the available analytical techniques.

The advent of gene microarrays permits the analysis of gene expression for thousands of genes simultaneously in biological samples of interest, permitting functional interpretation of the transcriptome state of any given cell type at a particular physiologic state [18,19]. These cellular mRNA expression profiles yield global genomic fingerprints that identify the biological state of that cell. Molecular profiling has rapidly become an effective approach to further understanding of the phenotypes of tumor cells [20,21]. Because the colony size of *Tk *mutants is determined by the growth rate of mutant cells, it would be expected that there would be some difference in the expression levels of one or more specific growth regulation genes. Microarray analysis should allow us to measure gene expression in large and small colony mutants and discern possible mechanisms that lead to the two phenotypes based on the differences between the gene expression profiles.

ArrayTrack software developed at the National Center for Toxicological Research provides an integrated solution for managing, analyzing, and interpreting microarray gene expression data. It is MIAME (Minimum Information about a Microarray Experiment) supportive for storing both microarray data and experiment parameters associated with a pharmacogenomics or toxicogenomics study. Using ArrayTrack, users can easily select a normalization method and a statistical method applied to a stored microarray dataset to cluster genes into different groups according to expression profiles, to determine a list of differentially expressed genes (significant genes), and to link the gene list directly to pathways and gene ontology for functional analysis. ArrayTrack is being integrated and further refined at the U.S. FDA as a review tool for the pharmacogenomics data submission program . ArrayTrack is also freely available to public [22].

In this study, previously isolated small and large *Tk *mutants were evaluated for differences in gene expression using microarray technology. We used this approach to address the following 4 questions: (1) whether microarray analysis using ArrayTrack could distinguish the two *Tk *mutant phenotypes; (2) whether microarray analysis could identify candidate genes that might contribute to the phenotype difference; (3) whether genes altered in their expression might be localized on chromosome 11 near the *Tk *gene; and (4) whether the results might provide insight into the underlying mechanisms responsible for the two types of mutants. For this analysis, we selected 4 small and 4 large colony mutant samples from a previously conducted 3'-azido-3'-deoxythymidine (AZT)-treatment [23]. These mutants were selected because they all showed the same microsatellite pattern. They were LOH for both *Tk *and D11Mit42 (see Figure 2). We used this strategy for the mutant selection so that we could determine if the microarray approach, which evaluates individual gene expression, would provide a level of resolution not provided by the microsatellite analysis. We found that microarray analysis using ArrayTrack distinguished the two different cellular phenotypes and identified a set of candidate genes that are responsible for the colony size phenotypes. Also, the expression of a high proportion of genes located near the *Tk *gene on chromosome 11 was differentially altered between the large and small colony mutant samples, which might be the original reason for the formation of two different sizes of colony mutants.

## Materials and Methods

### Cells and culture conditions

L5178Y/*Tk*^+/-^-3.7.2C mouse lymphoma *Tk*-deficient mutants were grown according to the methods described by Chen and Moore [4]. Briefly, the basic medium was Fischer's medium for leukemic cells of mice with L-glutamine (Quality Biological Inc., Gaithersburg, MD) supplemented with 10% heat-inactivated horse serum, pluronic F68 (0.1%), sodium pyruvate (1 mM), penicillin (100 U/ml), and streptomycin (100 μg/ml). The cultures were maintained in a humidified incubator with 5% CO_2 _in air at 37°C. Unless otherwise noted, all culture supplies were purchased from Invitrogen Life Technologies (Carlsbad, CA).

### Selection of large and small colony Tk mutants and expansion of mutant cells

**Figure 1:**
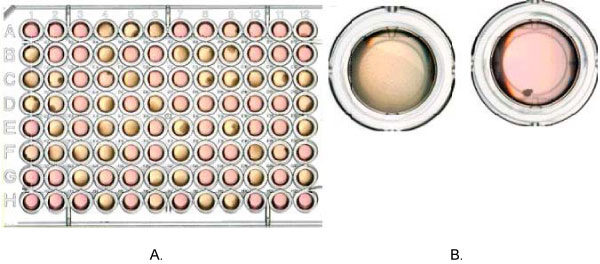
Large and small colony *Tk *mutants in TFT selective medium. A. shows different size of colonies in a 96-well plate and B. displays a typical large colony mutant and a typical small colony mutant.

**Figure 2:**
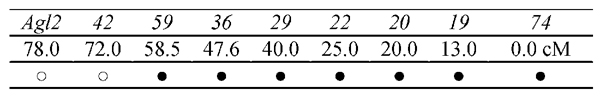
The loss of heterozygosity (LOH) pattern of all mouse lymphoma *Tk *mutants (4 small and 4 large colony mutant samples. The codes in the upper row are microsatellite loci on chromosome 11. The numbers in the middle row are the distances from the top of the chromosome to the locus in cM (centimorgan). The symbol ○ indicates LOH and ● indicates that the locus retains heterozygosity.

Large and small colony mutants were selected from a series of mutants isolated following AZT treatment [23]. An example of large and small colonies in selective medium in 96-well plates is shown in Figure 1. The mutants were selected based on the considerations described in the introduction. All mutants showed the same microsatellite LOH pattern (LOH at *Tk *and D11 Mit42 – see Figure 2) indicating that the chromosome alteration might occur at the *Tk *locus and extend up to 58.5 cM.

In addition, only mutants whose growth rates were relatively stable were chosen for microarray analysis of gene expression. The average doubling time for the large colony mutants was 9.6 ± 0.2 hours while the average doubling time for the small colony mutants was 17.5 ± 0.4 hours (*p *< 0.001, Student T-test).

### RNA Isolation and cDNA Labeling

Total RNA was extracted from mutants whose cells were collected at log stage of growth using Qiagen RNeasy kits with on-column DNase digestion. The concentration and quality of RNA samples were measured by a spectrophotometer and an Agilent 2100 Bioanalyzer (Agilent, Palo Alto, CA). All the RNA samples were stored at -80°C until used for microarray analysis.

The aminoallyl-indirect labeling protocol by The Institute for Genomic Research (TIGR, Rockville, MD) was followed with a few modifications. Briefly, 10 μg of total RNA was primed with 6 μg of random hexamer primers (Invitrogen, Carlsbad, CA) in a final volume of 16.5 μl. The RNA was reverse-transcribed in a 30 μl reaction containing 0.5 mM dATP, dCTP, dGTP, 0.3 mM dTTP (Invitrogen) and 0.2 mM aminoallyl-dUTP (aa-dUTP, Ambion), 40 U RNase inhibitor (Invitrogen), 400 U Superscript II (Invitrogen), 10 mM DTT and 1X first strand buffer at 42°C for 2 hours to generate aminoallyl-labeled cDNA. The purified aminoallyl-cDNA was coupled with either Cy3 or Cy5 monoreactive dyes (Amersham Pharmacia, Piscataway, NJ). Uncoupled dyes were removed by QIAquick PCR purification kit. cDNA yields and dye incorporation efficiencies were determined using a NanoDrop spectrophotometer. A reference design was used in which RNA sample from each large or small colony mutant was labeled with Cy5 and paired with Mouse Universal Reference RNA (Stratagene, La Jolla, CA) labeled with Cy3.

### Hybridization and imaging

Pairs of Cy3 and Cy5 labeled cDNAs were dissolved in 60 μl of hybridization buffer (5× SSC, 0.1% SDS with 27% formamide) and incubated with a mouse 20 k oligonucleotide microarray that was fabricated in-house at the NCTR using oligonucleotides from MWG, Inc. (High Point, NC). The hybridization was performed in hybridization cassettes (ArrayIt, Sunnyvale, CA) in a water bath at 50°C for 16–18 hours. The slides were then washed in pre-warmed (30°C) 2X SSC (containing 0.1% SDS) for 5 minutes, 1X SSC for 5 minutes, and 0.5X SSC for 5 minutes. The hybridized slides were dried immediately by centrifugation and scanned with a GenePix 4000B scanner (Axon Instruments, Sunnyvale, CA) at 10 μm resolution. The resulting images were analyzed by measuring the fluorescence of all features on the slides using the GenePix Pro 6.0 software (Axon Instruments). The median fluorescence intensity of all the pixels within one feature was taken as the intensity value for each feature.

### Data analysis

The raw data were imported into ArrayTrack (NCTR/FDA) and normalized using Total Intensity Normalization after subtracting backgrounds. Expression ratios (sample/universal RNA) from 8 arrays (4 large colony and 4 small colony mutant RNA samples) were log2-transformed. The logged data were used for principal component analysis (PCA), hierarchical cluster analysis (HCA), and statistical analysis for identifying genes that were differentially expressed in large versus small colony mutants. The Welch T-test was used for determining significant changes in gene expression between the large and small colony mutant groups. Significant genes were selected with a cut-off of *p *< 0.01 and fold change > 1.5. The selected genes were further analyzed for their functions and networks using Ingenuity Pathway Analysis (IPA, Ingenuity Systems Inc., Redwood City, CA). Information on genes' chromosome locations were obtained using Gene Library within ArrayTrack.

## Results

### Principal component analysis and hierarchical clustering of the gene expression profiles

**Figure 3:**
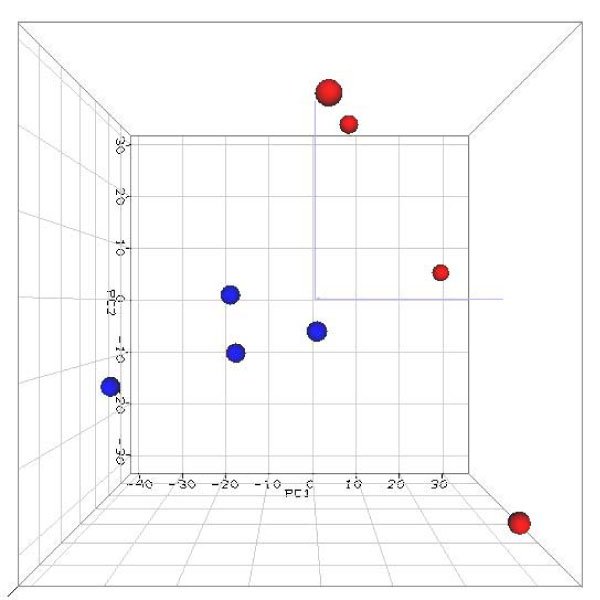
Principal component analysis (PCA) 3D view for gene expression profiles from 8 large and small colony *Tk *mutant samples. The PCA is based on log2 ratios and the expression profiles are across all the 20,000 genes in the microarrays. The blue and red dots indicate large and small colony mutants, respectively. The first three principal components are plotted. The captured variances of PC1 (first principal component), PC2 (second principal component) and PC3 (third principal component; the label is not shown) were 25.5%, 17.4%, and 15.5%, respectively.

**Figure 4:**
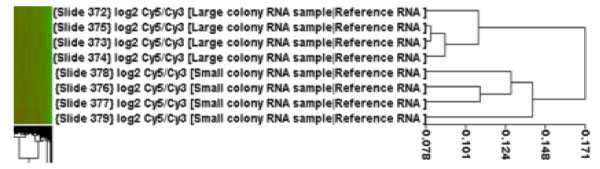
Hierarchical clustering of large and small colony mutant samples. Ward's Minimum Variance method was used. Clustering is based on log2 ratios and the expression profiles are across all the 20,000 genes in the microarrays.

Genome-wide expression profiles for 8 *Tk *mutant samples were generated using the mouse oligonucleotide microarray. Principal components analysis (PCA) within ArrayTrack was used to carry out an examination of the relationship among the samples (Figure 3). A separation between large and small colony mutants was clearly observed. The large colonies are more tightly grouped together while the small colonies more loosely grouped. Hierarchical Cluster Analysis (HCA) using ArrayTrack also revealed distinct grouping of the mutant samples according to their colony size, suggesting that gene expression patterns are different between the large and small colony mutants (Figure 4).

### Identification of differentially expressed genes between large and small colony Tk mutants

**Figure 5:**
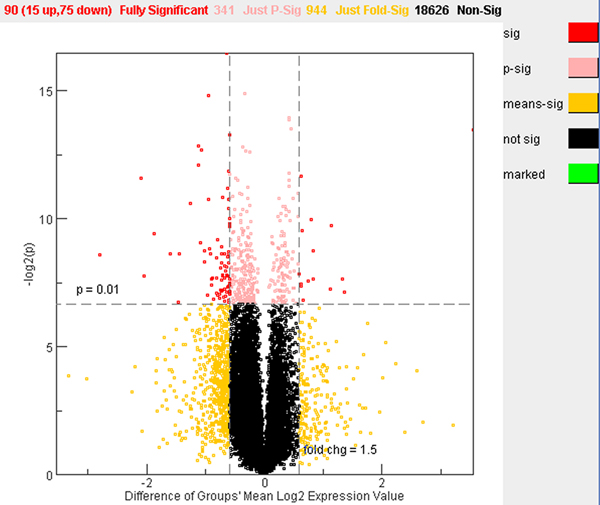
Volcano plot for differentially expressed genes between large and small colony mutants. The plot is based on log2 ratios and the expression profiles are across all the 20,000 genes in the microarrays. A gene is identified as significantly altered if the *p*-value is smaller than 0.01 and fold change is greater than 1.5. Red dots indicate significant genes; pink dots indicate genes that have *p *< 0.01 and fold changes < 1.5; yellow dots indicate genes that have *p *> 0.01 and fold change > 1.5; and black dots indicate genes that have *p *> 0.01 and fold change < 1.5.

Welch T-test was used for determining significant changes in gene expression between large and small colony mutant groups. A volcano plot for differentially expressed genes between large and small colony mutants is shown in Figure 5. A group of 90 genes was found to be differentially expressed with a cutoff (*p *< 0.01 and fold change > 1.5). Among the 90 genes, 15 genes were up-regulated and 75 genes were down-regulated when the gene expression ratios in large colony mutants were compared to those in small colony mutants (large/small). A complete list of these genes can be found in Table 1. There were 431 genes whose *p*-value was smaller than 0.01 without fold-change cutoff and 1034 genes that have a fold-change greater than 1.5 without considering the *p*-value.

### Pathway analysis of the significant genes

The selected genes were analyzed for their functions using IPA that is integrated with ArrayTrack. Among the 90 genes, 50 genes were mapped to the IPA database and were used for functional analysis. The 50 genes were mapped to four different networks in the IPA database (Table 2). The scores for all of the mapped networks were higher than 19, indicating that the networks selected were not due to random chance alone. (A score of 3 or greater was considered significant at *p *< 0.001 level). These networks were associated with the following pathways: gene expression, cell signaling, lymphatic system development and function, cellular growth and proliferation, cell death and cancer.

### Significant genes located on chromosome 11

**Figure 6:**
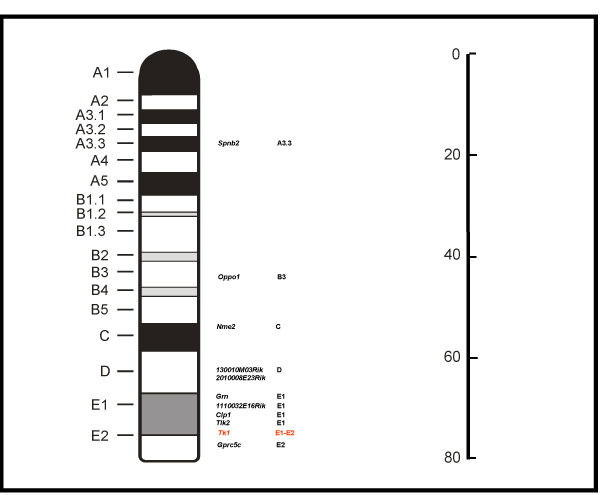
Map for chromosome 11 genes whose expression was significantly altered. The position of *Tk1 *gene in red is also shown for comparison. The right ruler indicates distance in cM (centimorgan) from top of the chromosome.

The mouse lymphoma mutants are identified and isolated because they have mutation(s) that involve the *Tk *gene that is located at the distal end of chromosome 11. The mutation can occur solely within the *Tk *gene or it may also involve other genes on chromosome 11. While the underlying difference between the small and large colony mutants has remained elusive, it is clear that the slow growth of the small colony mutants must be caused by an alteration in the expression of one or more genes related to cell growth. It is expected that one or more genes located near the *Tk *gene on chromosome 11 may have differential expression in large and small colony mutants because of an alteration of the DNA in the region around the *Tk *gene. The Gene Library feature of ArrayTrack was used to identify the significant genes located on chromosome 11. Among 90 significant genes, 10 of them were found to be located on chromosome 11 (about 11%). Interestingly, all these genes had a higher gene expression in small colony mutants than in large colony mutants (Table 1 and Figure 6). Among the total genes (20,000) in the array, about 5.6% of them are located on chromosome 11 and no bias for up or down-regulation of genes was found.

## Discussion

The large and small colony *Tk *mutants differ in their growth kinetics. The cells from the small colony mutants grow slowly while the cells from large colony mutants grow at normal rates [5,9]. The relative proportion of these two classes of mutants is mutagen dependent and relates to the clastogenic, anuploidogenic and recombinogenic potential of the chemical. Despite extensive molecular genetic and cytogenetic evaluation of a large number of mutants, the fundamental mechanistic difference(s) between the two phenotypes is not known. To explore the biological difference between the two types of *Tk *mutants, four large colony mutant samples with an average doubling time of 9.6 ± 0.2 hours and four small colony mutant samples with an average doubling time of 17.5 ± 0.4 hours were examined by microarray gene expression analysis.

PCA and HCA of the gene expression profiles from these mutant samples showed that the samples were clearly separated into two groups based on their phenotype of colony sizes. Given that both PCA and HCA were conducted using all of the genes on the microarray (20K) without filtering, this finding is very significant, indicating a high quality of the microarray experiment and strong biological relevance. The results can be interpreted as evidence for the biological similarity within each size group and biological differences between the two different size colony mutants. Also the results from PCA (Figure 3) showed that the gene expression patterns in the large colony group of mutants were very homogeneous while the expression patterns from the small colony group of mutants were relatively heterogeneous. These patterns reflect the variability of the colony size in the two groups. Generally, large colony mutants are similar in size to wild-type colonies; the growth rate of these cells is also similar. The growth rate of cells in the small colony mutants is slower and more variable than wild-type cells. The standard deviation of the growth rate of cells from the small colony mutant samples was greater than that for cells from the large colony mutants.

Expression of 90 genes was significantly altered between large and small colony mutants. When comparing the expression of large colony mutants to small colony mutants, 15 genes were up-regulated and 75 genes were down-regulated. This bias might result from the characteristics of these genes. Most of these genes are involved in regulation of growth. Twenty-two of 50 genes mapped into IPA database were involved in the regulation of apoptosis. They are Cdh2, Cdkn2A, Chrm1, Cspg2, Ets1, Fgfr1, Gata6, Grn, Ifi202b, Il17rd, Irf2, Lamb2, Neu3, Nppa, Pdgfra, Ptn, Ptprc, Rag2, Sema3b, Stat1, Tgfbr2, and Tp53inp1. Expression of 18 genes among them was higher in small colony mutants than large colony mutants. This pattern of expression suggests that more apoptosis occurred in the small colony mutants, leading to the slow growth of the cells.

Consistent with the growth rate phenotype is the finding that nearly all significant genes that can be found in IPA database were mapped into networks that were related to cell growth (see Table 2).

Genes of particular interest in this group are recombination activating gene 2 (Rag2) and cyclin-dependent kinase inhibitor 2A (Cdkn2a). Because a large portion of *Tk *mutants may result from recombinational events, we have proposed that the mouse lymphoma cells have a high recombinase activity. The Rag1 and Rag2 genes are necessary for V(D)J recombination [24,25]. Also, Rag2 is necessary for lymphocyte maturation and growth [26,27]. A 2.21-fold lower activity of Rag2 gene expression in small colony mutant (Table 1) would restrict the cell growth of small colony mutants. Because the large colony cells grow at a similar growth rate as the wild type mouse lymphoma cells, the high expression of Rag2 in large colony cells might suggest that mouse lymphoma cells have an elevated level of the recombinase activity and these cells might be particularly sensitive to recombination and therefore particularly effective in detecting chemicals that induce recombination. Cdkn2a is known to be an important tumor suppressor gene [28] and generates several transcript variants that function as inhibitors of CDK4 kinase [28,29]. This protein also sequesters MDM1, a protein involved in the degradation of p53, and thus may serve to stabilize p53 [30]. Therefore, this protein functions in cell cycle control and it is frequently mutated or deleted in a wide variety of tumors [31-35]. A 2.02-fold higher expression activity of this tumor suppressor gene in small colony mutants (Table 1) would favor a slower growth of those cells than the large colony cells.

Of particular interest, the significant genes were disproportionally distributed in the vicinity of the *Tk *gene on chromosome 11 with a higher expression level in small mutant colony mutants than in large colony mutants. Ten of the significant genes were located on chromosome 11. However, if the genes were distributed on the chromosomes proportionally, there would have been about 2 genes with up-regulation of expression in the small colony mutants on chromosome 11. Moreover, 7 of the 10 genes were located within 20 cM of the *Tk *gene (Figure 6). This gene distribution pattern may be explained by chromosomal location rather than by gene function, suggesting a regional change in gene expression. Further, this suggests that the type of DNA alteration that inactivates the *Tk *gene in the small colony mutants is responsible for this regional change in gene activity. Although the microsatellite LOH pattern in the D and E bands of chromosome 11 (Figure 2 and Figure 6) was the same for all the mutants, the gene expression in this region appeared significantly different between the large and small colony mutants.

## Conclusion

The PCA and HCA showed that the gene expression profiles from the mutant samples were clearly separated into two groups based on their colony sizes. This cluster pattern indicates biological similarity within each mutant group and biological difference between the two different colony size mutants. Statistical and functional analysis of the profiles identified a set of genes whose expression was differentially altered between large and small colony mutants. Most of these genes are responsible for regulation of cell growth that would be expected to influence the cell growth rate and the colony sizes. In addition, we found a number of significant genes that were disproportionally concentrated on an area of chromosome 11 where there was loss of heterozygosity. These findings suggest that the *Tk *chromosome mutations in the small *Tk *colony mutants result in alterations in gene expression for genes located near the *Tk *gene. This same altered gene expression does not occur in the large colony mutants. This finding is particularly interesting because the microsatellite LOH analysis indicates no difference between the small and large colony mutants. Our analysis demonstrates that the utility of microarray analysis using ArrayTrack provides a gene level analysis of the two cellular phenotypes. Using this tool, we have identified genes that appear to be related to the colony size phenotype. The analysis of additional mutants would be expected to provide further information and may elucidate the underlying difference between the small and large colony *Tk *mutants.

## Authors' contributions

TH performed the experiments for generating the original data and was involved in writing the manuscript. JCF and TH did the original microarray data analysis. JW generated the mutant cells; tested the genotypes of the mutants; isolated the mRNA from the mutants; and was involved in the experimental design. WT, MMM and JCF participated in the overall design of the study, discussion of the data analysis, and assistance with writing the manuscript. TC had the original idea for this study, performed the data analysis, and wrote the manuscript. All authors approved the final manuscript.

## Figures and Tables

**Table 1:** Genes differentially expressed between large and small colony mutants (*p *< 0.01; fold change > 1.5).

Locus ID	Gene Name	*P *Value	Fold Change	Description
109815	*H47*	0.0000	-1.55	histocompatibility 47
93707	*Pcdhgc4*	0.0000	-1.93	protocadherin gamma subfamily C, 4
100121	*5730495N10Rik*	0.0001	11.68	tudor domain containing 7
23936	*Lynx1*	0.0001	-1.51	Ly6/neurotoxin 1
74558	*9130002C22Rik*	0.0001	-2.18	GTPase, very large interferon inducible 1
77705	*9230104L09Rik*	0.0002	-2.10	RIKEN cDNA 9230104L09 gene
18028	*Nfib*	0.0002	-2.19	nuclear factor I/B
14081	*Acsl1*	0.0003	1.55	acyl-CoA synthetase long-chain family member 1
12669	*Chrm1*	0.0003	-1.53	cholinergic receptor, muscarinic 1, CNS
14824*	*Grn*	0.0003	-4.26	granulin
60599	*Trp53inp1*	0.0004	-1.54	transformation related protein 53 inducible nuclear protein 1
268490*	*1110032E16Rik*	0.0006	-1.53	RIKEN cDNA 2600001B17 gene
68473	*1110003E08Rik*	0.0006	-1.63	MOB1, Mps One Binder kinase activator-like 1A (yeast)
16391	*Isgf3g*	0.0006	-1.93	interferon dependent positive acting transcription factor 3 gamma
14182	*Fgfr1*	0.0008	-1.53	fibroblast growth factor receptor 1
71723	*Dhx34*	0.0010	1.73	DEAH (Asp-Glu-Ala-His) box polypeptide 34
494468	*MGC38735*	0.0010	-1.51	hypothetical protein
20742*	*Spnb2*	0.0011	-1.50	spectrin beta 2
19374	*Rag2*	0.0012	2.21	recombination activating gene 2
18103*	*Nme2*	0.0012	-1.50	expressed in non-metastatic cells 2, protein
66359	*2310005N03Rik*	0.0014	1.56	RIKEN cDNA 2310005N03 gene
194367		0.0015	-3.69	Not found
72700	*2810040O04Rik*	0.0017	-1.73	Zinc fingerprotein 618
231293	*C130090K23Rik*	0.0019	-2.14	RIKEN cDNA C130090K23 gene
27660	*1700088E04Rik*	0.0021	-1.66	RIKEN cDNA 1700088E04 gene
55932	*Gbp3*	0.0022	-1.91	guanylate nucleotide binding protein 4
19245	*Ptp4a3*	0.0024	1.77	protein tyrosine phosphatase 4a3
17722	*Nd6*	0.0024	-1.53	NADH dehydrogenase subunit 6
12578	*Cdkn2a*	0.0024	-2.02	cyclin-dependent kinase inhibitor 2A
20846	*Stat1*	0.0026	-1.52	signal transducer and activator of transcription 1
77015	*2700082O15Rik*	0.0026	-1.61	metallophosphoesterase domain containing 2
50877	*Neu3*	0.0026	-1.65	neuraminidase 3
67998*	*1300010M03Rik*	0.0026	-1.88	RIKEN cDNA 1300010M03 gene
171463	*Il17rd*	0.0026	-2.72	interleukin 17 receptor D
20363	*Sepp1*	0.0026	-3.05	selenoprotein P, plasma, 1
12833	*Col6a1*	0.0026	-6.94	procollagen, type VI, alpha 1
13612	*Edil3*	0.0028	-1.54	EGF-like repeats and discoidin I-like domains 3
77773	*A330103N21Rik*	0.0028	-1.77	RIKEN cDNA A330103N21 gene
228543	*Arhv*	0.0032	-2.04	ras homolog gene family, member V
230899	*Nppa*	0.0033	-1.52	natriuretic peptide precursor type A
3337208	*Cytb*	0.0033	-1.65	cytochrome b
12558	*Cdh2*	0.0037	-1.57	cadherin 2
192231*	*Clp1*	0.0037	-1.67	hexamethylene bis-acetamide inducible 1
93875	*Pcdhb4*	0.0041	-1.58	protocadherin beta 4
72080	*2010317E24Rik*	0.0044	1.51	RIKEN cDNA 2010317E24 gene
27360	*Add3*	0.0045	-1.57	adducin 3 (gamma)
14431	*Gamt*	0.0046	-1.65	guanidinoacetate methyltransferase
13841	*Epha7*	0.0047	-1.55	Eph receptor A7
21356	*Tapbp*	0.0047	-1.63	TAP binding protein
26388	*Ifi202b*	0.0047	-4.15	interferon activated gene 202B
77974	*Rdh12*	0.0050	-1.60	retinol dehydrogenase 12
11542	*Adora3*	0.0050	-1.76	adenosine A3 receptor
16909	*Lmo2*	0.0050	-1.84	LIM domain only 2
16779	*Lamb2*	0.0051	2.50	laminin, beta 2
19264	*Ptprc*	0.0051	1.78	protein tyrosine phosphatase, receptor type, C
57754	*1500001H12Rik*	0.0051	-1.50	RIKEN cDNA 1500001H12 gene
225849	*Ppp2r5b*	0.0051	-1.88	protein phosphatase 2, regulatory subunit B (B56), beta isoform
15950	*Ifi203*	0.0052	-1.65	interferon activated gene 203
258568	*MOR202-20*	0.0053	1.68	olfactory receptor 1457
23871	*Ets1*	0.0058	1.54	E26 avian leukemia oncogene 1, 5' domain
21813	*Tgfbr2*	0.0058	-1.50	transforming growth factor, beta receptor II
65116	*Prrg2*	0.0060	-1.52	proline-rich Gla (G-carboxyglutamic acid) polypeptide 2
192231	*Clp1*	0.0060	-1.88	hexamethylene bis-acetamide inducible 1
20641	*Snrpd1*	0.0062	1.54	small nuclear ribonucleoprotein D1
252868*	*Oppo1*	0.0062	-1.52	outer dense fiber of sperm tails 4
380832	*Tcrg-C*	0.0066	2.17	T-cell receptor gamma, constant region
24086*	*Tlk2*	0.0067	-1.61	tousled-like kinase 2 (Arabidopsis)
67775	*5830458K16Rik*	0.0068	-1.64	RIKEN cDNA 5830458K16 gene
15042	*H2-T24*	0.0069	-1.68	histocompatibility 2, T region locus 24
24066	*Spry4*	0.0071	-1.51	sprouty homolog 4 (Drosophila)
15425	*Hoxc6*	0.0071	-1.65	homeo box C6
14934	*Gypa*	0.0074	2.57	glycophorin A
230587	*Gli6*	0.0074	-1.55	GLIS family zinc finger 1
19242	*Ptn*	0.0074	-1.77	pleiotrophin
211770	*A530090O15Rik*	0.0077	-1.51	tribbles homolog 1 (Drosophila)
20128	*Trim30*	0.0077	-1.70	tripartite motif protein 30
22441	*Xlr*	0.0079	-1.73	X-linked lymphocyte-regulated complex
66205	*1110055L24Rik*	0.0079	-1.96	CD302 antigen
70110*	*2010008K16Rik*	0.0081	-1.58	interferon-induced protein 35
214855	*D430024K22Rik*	0.0084	-1.62	AT rich interactive domain 5A (Mrf1 like)
13003	*Cspg2*	0.0086	-1.70	chondroitin sulfate proteoglycan 2
70355*	*Gprc5c*	0.0087	-1.82	G protein-coupled receptor, family C, group 5, member C
67974	*5730405I09Rik*	0.0089	1.58	RIKEN cDNA 5730405I09 gene
20347	*Sema3b*	0.0091	-1.53	sema domain, immunoglobulin domain (Ig), short basic domain, secreted, (semaphorin) 3B
16364	*Irf4*	0.0092	-1.60	interferon regulatory factor 4
18595	*Pdgfra*	0.0095	-2.77	platelet derived growth factor receptor, alpha polypeptide
14465	*Gata6*	0.0097	-1.53	GATA binding protein 6

Note: Three unknown genes are not included. * indicates the gene is located on chromosome 11.

**Table 2:** List of Ingenuity networks generated by mapping the focus genes that were differentially expressed between large and small colony mutants.

Network	Genes*	Score^#^	Focus Genes	Top Functions
1	**Add3**, **Adora3**, Agt, Asf1b, Cdkn1b, **Cdkn2a**, Cks1b, **Col6a1**, Col6a2, **Cspg2**, Ctcf, Cyp11b2, Egfr, Eps8, Erbb2, Fbn1, **Fgfr1**, **Gbp4**, Gpc1, Hmga2, **Il17rd**, **Irf4**, Mell1, Nfatc2, **Nfib**, **Nppa**, Npr3, P4ha2, Ptrf, **Rag2**, **Tgfbr2**, **Tlk2**, Top1, **Tp53inp1**, Wap	25	15	Cellular Growth and Proliferation, Cancer, Cell Death
2	**Acsl1**, Angpt1, Btg1, Btg2, Ccnt1, Ccnt2, Cdk9, Cebpa, **Chrm1**, Dgkz, Efna1, Elf1, **Epha7**, Fbl, **Grn**, **His1**, **Ifi203**, **Lamb2**, Mapk1, Mmp17, Myc, **Nme2**, Oas1, **Ptprc**, Ptx3, Rasgrp1, Rn7sk, Rock1, Slpi, **Spry4**, Sytl1, **Tapbp**, **Tdrd7**, Tnf, **Trib1**	21	13	Gene Expression, Cellular Development, Cell-To-Cell Signaling and Interaction
3	Alox15, Btg1, Cdkn1a, Cks1b, **Col6a1**, Col6a2, Col6a3, Ctcf, Ebi3, Fgf13, **Gata6**, **Gvin1**, **Ifi35**, **Ifi202b**, Ifna, Ifne1, Ifng, Il15, Il12rb1, Il17f, Il27ra, **Isgf3g**, Mylk, **Nd6**, Ndufs4, Pdgfc, **Pdgfra**, S100a11, **Sema3b**, **Sepp1**, **Stat1**, Stat4, Tgfb1, **Trim30**, Znf467	19	12	Cancer, Cell Death, Cellular Growth and Proliferation
4	Casp3, Cckbr, Cd1d, **Cdh2**, Ctcf, Ctnna2, Ctnnb1, Ctnnd2, Cyp24a1, **Edil3**, **Ets1**, **Gypa**, Il2, Invs, **Lmo2**, Mdm4, Mgat5, **Neu3**, Nf2, Pag, Pitx2, Ppp2ca, Ppp2cb, Ppp2r2c, **Ppp2r5b**, **Ptn**, **Ptp4a3**, **Ptprc**, Scn5a, Slc4a1, **Snrpd1**, **Sptbn1**, Tbxas1, Tp53, Tuba1	19	12	Cellular Development, Hematological System Development and Function, Immune and Lymphatic System Development and Function

Note: * Bold genes are those mapped by the significant genes. ^# ^A score of 3 or greater was considered significant (*p *< 0.001)

**Table 3:** Location and function of the significant genes located on chromosome 11.

Gene Name	Location	Gene product and function
*1110032E16Rik*	E1	Hypothetical protein LOC268490 with unknown function
*1300010M03Rik*	D	Hypothetical protein LOC67998 with unknown function
*2010008K16Rik*	D	Interferon-induced protein with unknown function
*Clp1*	E1	Cleavage and polyadenylation factor CF I component involved in pre-mRNA 3'-end processing
*Gprc5c*	E2	G protein-coupled receptor, family C, group 5, member C with unknown function
*Grn*	E1	Granulin that acts as inhibitors, stimulators, or have dual actions on cell growth.
*Nme2*	C	Expressed in non-metastatic cells 2, protein involves in cell proliferation.
*Oppo1*	B3	Outer dense fiber of sperm tails 4 with unknown function.
*Spnb2*	A3.3	Spectrin involving disruption, proliferation of cells.
*Tlk2*	E1	Tousled-like kinase 2 that function as regulation of chromatin assembly or disassembly; protein amino acid phosphorylation; response to DNA damage stimulus; cell cycle; intracellular signaling cascade; chromatin modification
